# Sensitivity to losses and defects of the symmetry-induced transmission enhancement through diffusive slabs

**DOI:** 10.1038/s41598-020-73701-0

**Published:** 2020-10-06

**Authors:** Élie Chéron, Simon Félix, Vincent Pagneux

**Affiliations:** Laboratoire d’Acoustique de l’Université du Mans (LAUM), UMR 6613, Institut d’Acoustique - Graduate School (IA-GS), CNRS, Avenue Olivier Messiaen, 72085 Le Mans, France

**Keywords:** Acoustics, Physics

## Abstract

We inspect the robustness to absorption and to symmetry defects of the symmetry-induced broadband enhancement through opaque barriers in disordered slabs. The sensitivity of this phenomenon to symmetry defects is found to be strongly related to the distance from to barrier to the nearest defect, and, following, we propose a probabilistic model to estimate the conductance of a medium with an arbitrary number of randomly distributed defects. Also, the conductance enhancement is shown to be robust to absorption in the disordered medium, though being of course weakened. For sufficiently opaque barriers, the conditions of an optimal enhancement are mainly driven by the absorption length of the medium.

## Introduction

Although the diffusion model is well fitted to describe wave propagation in disordered media, it neglects that waves, which follow numberless trajectories, can return to the same coherent volume, giving rise to important interference effects, such as conductance fluctuations, enhanced backscattering or Anderson localization^1–4^. Remarkable consequences of multiple interferences can also be observed in complex media when the scattering region displays symmetries^5–11^, and the authors recently reported on a significant broadband enhancement of the transmission through an a opaque barrier in a waveguide, when placed between symmetric diffusive slabs^12^.

The question then naturally arises as to the robustness of this transmission enhancement to absorption and to symmetry defects. The last point was partially answered in^12^, showing a strong sensitivity when moving an increasing number of randomly chosen scatterers in the slabs on either side of the barrier. This is further investigated in the present paper, linking the degree of sensitivity to symmetry defects to the position of these defects. We numerically show that the distance from the barrier to the nearest defect is the determining parameter, rather than the amount of defects. From this observation, we propose a probabilistic model to estimate the conductance of a medium with a number of randomly distributed defects.

As for absorption, ubiquitous in all acoustical or optical media where we would expect to observe the symmetry-induced transmission enhancement, it is all the more important to characterize its influence as it often complicates, if not compromises, the chance of experimental evidence of certain wave-based phenomena^13–15^. Typically, absorption (even weak^16^) can strongly alter the open channels, hence the transmission, through a disordered medium^17,18^. The robustness of the transmission enhancement to losses is investigated in a second part of this paper by introducing absorption in the disordered slabs on either sides of the opaque barrier. We show that the enhancement, while being of course a little bit weakened, still occurs, and it is then mainly driven by the absorption length of the medium.

**Figure 1:**
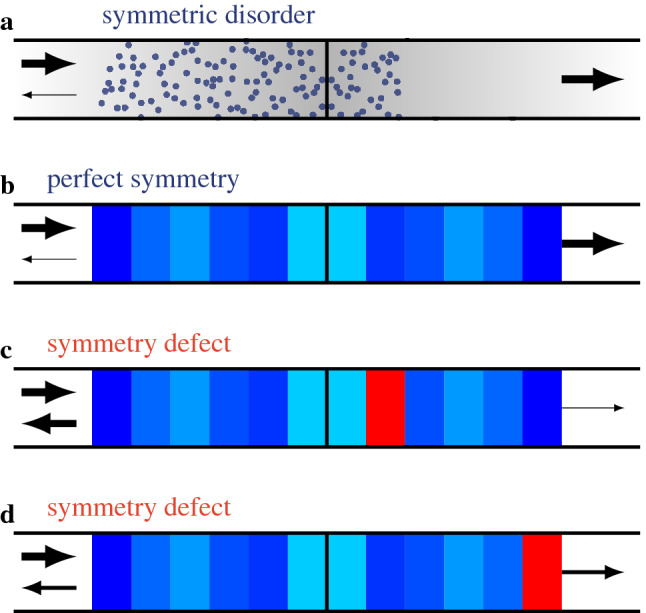
Sketches of the studied scattering system with a symmetry defect. **(a)** The original system is a symmetric “slab-barrier-slab” (SBS) system, and the diffusive slabs are made of a random distribution of scatterers. **(b)** The scattering by the SBS within a waveguide is solved using the random matrix theory (RMT), and the slab are then considered as being composed as *M* $\ell$-length layers ($\ell$ the mean free path), the scattering matrix of which is a COE random matrix. Then, from the fully symmetric configuration shown in **(b)**, the symmetry is broken by modifying one layer (in red), which can be either **(c)** close to or **(d)** far from the barrier.

The system under study is a quasi-one-dimensional waveguide that supports *N* propagating modes. The refractive index of a given region in the waveguide is perturbed to account for the presence of randomly distributed scatterers, and a thin opaque barrier splits this region into two equal-length slabs. Besides, in order to observe the transmission enhancement, one imposes the spatial perturbation $\delta n(x,y)$ of the refractive index to obey a left-right mirror symmetry with respect to the barrier (Fig. 1a). In each slab the propagation is governed by the heterogeneous Helmholtz equation

1 $$\begin{aligned} \Delta {\psi (x,y)} + k^2 \left[ 1 + \delta n(x,y) \right] ^2 \psi (x,y) = 0, \end{aligned}$$

and, beside the wavelength $\lambda = 2\pi /k$, the relevant length scales are the length *L* of the disordered region and the mean free path $\ell$. Let $s\equiv L/\ell$ be the length of the scattering region in mean free path unit; we assume that $1 \ll s\ll N$, so that the incident flux is diffusively transmitted through the slabs. The conductance of the “slab-barrier-slab” (SBS) system within the waveguide is computed as given by the Landauer formula $g = Tr (\textsf {T}\textsf {T}^\dagger )$, $\textsf {T}$ the transmission matrix of the propagating modes^19^. Note that, in the absence of a barrier and under the assumption of diffusive transport, the conductance fulfills the classical Ohm’s law $g = N/(1+s)$^1,20,21^, allowing thus a direct determination of the mean free path $\ell$ from the numerical computations.

## Symmetry breaking

The initial configuration we consider is the optimal configuration, in which the maximum conductance enhancement is reached. In this configuration the length of the symmetric SBS is, in mean free path unit,

2 $$\begin{aligned} s_opt = \sqrt{\left( \frac{1}{\tau }-1\right) \left( \frac{1}{\tau _c }-1\right) } - \left( \frac{1}{\tau _c }-1\right) , \end{aligned}$$

where $\tau$ is the reduced transmittance of the barrier, defined such that a perfectly opaque barrier has a transmittance $\tau = 0$ and a perfectly transparent barrier has a reduced transmittance $\tau = 1$ corresponding to a perfect transmission. The formula above, however, is valid in a narrower range, since a threshold strength is required to achieve a conductance enhancement, namely $\tau < \tau _c$. This threshold value, deduced as Eq. (2) from a scaling model (cf. “A scaling model for the scattering with absorption”) and numerical data, is found to be $\tau _c \simeq 0.4$. In this optimal configuration, the conductance reaches a maximum value $g_opt$, and any symmetry defect, due to a shift of the barrier position^22^ or to a change in the spatial distribution of the scatterers, will result in a decrease of the conductance. To get insight of the sensitivity to symmetry breaking, let us see how it is related to the distance from the barrier to the nearest defect.

Throughout the paper, unless otherwise stated, the scattering by the diffusive slabs on either side of the barrier is solved using random matrices, as this approach has proven to be relevant and effective^12,23^. Each slab is considered as being composed of *M* layers whose length is the mean free path $\ell$. The scattering matrix of each layer is modeled by elements of the Dyson circular orthogonal ensemble (COE), to fulfill the properties of reciprocity (symmetry) and energy conservation (unitarity), and the scattering matrix of the slab is then constructed iteratively from the *M* layer matrices. Finally, once one slab has been “built,” the scattering matrix of its symmetric counterpart is straightforwardly deduced from this first operation. To account for symmetry defects, the scattering matrix of *n* among the *M* layers of one slab are replaced by new randomly generated matrices, and the distance (in mean free path unit) from the barrier to the nearest defect is denoted as $d \in [0, M-1]$ (Fig. 1).

**Figure 2:**
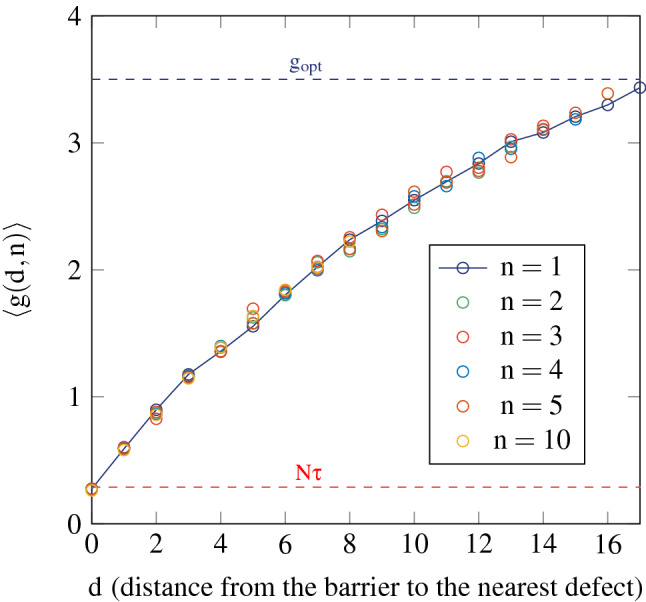
Conductance (averaged over 100 iterations) of the “slab-barrier-slab” (SBS) system as a function of the distance from the barrier to the nearest defect, and for several values of *n*, the number of modified, asymmetric, layers. Each slab is composed of $M = 25$ layers. Red dashed line: transmittance $N\tau$ of the barrier. Blue dashed line: optimal conductance $g_opt$ of a fully symmetrical system, with each slab having a length $s_opt$, see Eq. (2).

Figure 2 shows the dependence of the conductance of the SBS with *d* and the number of modified layers, *n*. It clearly reveals that the limiting factor for a conductance enhancement is the distance from the barrier to the nearest defect, rather than the “amount” of defects, as could suggest our first observations^12^. Whatever the number of defects, if the nearest one is directly adjacent to the barrier ($d = 0$), then there is no enhancement: the conductance value is the one of a fully non-symmetrical system, slightly smaller than $N\tau$, the transmittance of the barrier (red dashed line). Conversely, the transmission through the system is only slightly perturbed if the nearest defect is far enough from the barrier, and the observed conductance is close to its optimal value (blue dashed line). More generally, the evolution of the average conductance with *d* can be described with a function *G*(*d*) independent of *n*, given, for example, by computing the case with a single defect ($n = 1$).

**Figure 3:**
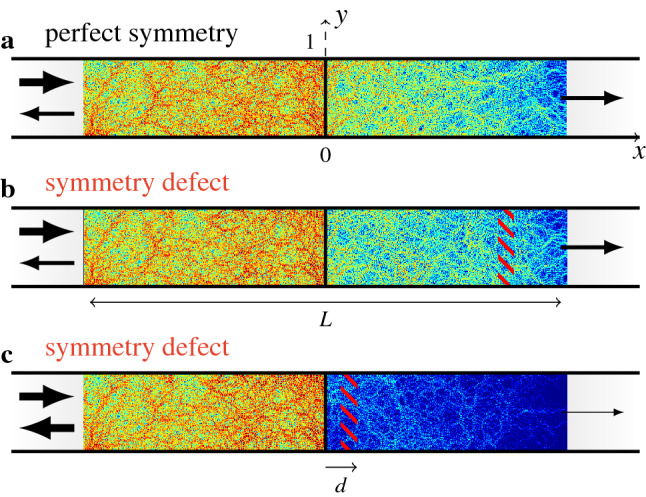
Typical wavefield (full wave numerical computation) in the SBS, when impinged by incident unit fluxes on each of the *N* propagating modes in the left lead ($N = 50$, $\ell = 0.46$, $L = 6$). **(a)** Fully symmetrical system. **(b)** Same as **(a)**, with symmetry defects far from the barrier. **(c)** Same as **(a)**, with symmetry defects close to the barrier.

These results are confirmed and illustrated in Fig. 3, which shows full wave numerical computations^24–26^ of the wave field in three cases: (a) a fully symmetrical system, (b) the same configuration, with symmetry defects far from the barrier, and (c) the same configuration, with symmetry defects close to the barrier. While the wavefield is only slightly perturbed in case (b), the overall level behind the barrier is much lower in case (c), denoting a strong decrease of the conductance.

**Figure 4:**
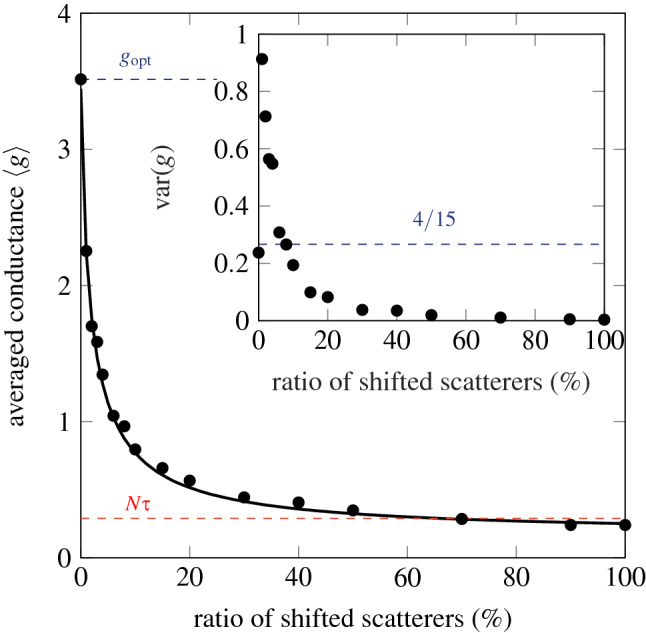
Decrease of the averaged conductance when shifting an increasing number of scatterers from the initially symmetric configuration. Plain circles: full wave numerics, solid line, Eq. (4). The red horizontal dashed line shows the barrier transmittance $N \tau$, while the blue dashed line shows the conductance provided the perfectly symmetrical configuration. The frequency is such that $N = 300$. The mean free path in the diffusive slabs is $\ell = 0.14$ in waveguide width unit, the length of the SBS is $L = 5$ and the sizes of defect layers are $\ell /5.6$. Inset: variance of the conductance. It decreases monotonically when increasing the number of shifted scatterers. Highlighted by the blue dashed line is the universal conductance fluctuation in disorder slabs, raised by a factor of two due to the mirror symmetry^6,9,27^.

Let us now take a look at the decrease of the average conductance when an increasing number of randomly chosen scatterers are shifted inside the slab, no matter their distance from the barrier^12^. A full wave numerical computation shows the rapid decrease in conductance with the ratio of shifted scatterers, that is, the amount of symmetry defects (Fig. 4). Given the result above (Fig. 2), this observation may be simply recovered, hence explained. As before, we start from a left-right symmetric distribution of 2*M* layers, with the barrier as symmetry axis. Then we randomly choose *n* among the *M* layers of one slab, and change their scattering matrix to a new one, to break the symmetry. *M* and *n* being known, the probability of finding a modified layer at a distance *d* from the barrier can be calculated. It reads

3 $$\begin{aligned} P(n,d) = {\left\{ \begin{array}{ll} \dfrac{(M-1-d)!(M-n)!n}{(M-n-d)!M!} &{} if M \ge n+d,\\ 0 &{}\text { otherwise.} \end{array}\right. } \end{aligned}$$

In particular, a single defect has an evenly distributed probability of being at a distance *d* from the barrier: $P(1,d) = 1/M$.

Finally, when averaging over a large number, the conductance, as a function of the number of defects, can be deduced from *P*(*n*, *d*) and the position-dependent conductance *G*(*d*) as

4 $$\begin{aligned} g(n) = \sum \limits _{d=0}^{M-1} P(n,d) \, G(d). \end{aligned}$$

This simple counting (*P*), once the dependency to *d* is known for, e.g., a single defect (*G*), allows us to recover accurately the result shown in Fig. 4 from the full wave numerics.

The function *P*(*n*, *d*) is a non-zero function if $M \ge n+d$. Consequently, increasing the number *n* of defects in a *M*-layer slab decreases the variance of the averaged conductance. In Fig. 4 inset we show that the variance $\text {var}(g)$ decreases monotonically when increasing the number of shifted scatterers. Note that the universal conductance fluctuation $\text {var}(g) = 2/15$, given by the DMPK equation in diffusive regime, is raised by a factor two in there is a perfect left-right symmetry^6,9,27^.

## Enhanced transmission through absorbing symmetric slabs

Absorption, ubiquitous in either acoustical or optical systems, must not only be taken into account to accurately describe and interpret experiments, but also because, more fundamentally, it may significantly affect the transport properties, starting with the regimes of transport themselves^28^. Thus, regarding the effect of symmetry we are interested in, we may wonder about the fate of the observed enhancement when absorption occurs. The following section shows how absorption modifies, and may even predominantly drive, the level of enhancement. First, we give reminders on absorbing diffusive transport in a slab. Then a scaling model is proposed for the symmetric SBS, that generalizes the model that what proposed in Ref.^12^ and inspired by Ref.^29^.

### Transmission through absorbing diffusive slabs

Let us consider first the disordered waveguide without opaque barrier. To account for a loss mechanism in the disordered slabs, we simply add an imaginary part to the refractive index the Helmholtz equation (1):

5 $$\begin{aligned} \Delta {\psi (x,y)} + k^2 \left[ 1 + \delta n(x,y) + i \gamma \right] ^2 \psi (x,y) = 0. \end{aligned}$$

In practice, the absorption could result from different loss mechanisms, in the scatterers, in the background medium, or at the waveguide walls. It would require a heterogeneous imaginary part $\gamma (x,y)$, inducing additional scattering. For the sake of simplicity, we consider $\gamma$ as being uniform in the slab, thus simply inducing a damping of the wave, noting that a more complex case with heterogeneous absorption leads to the same conclusions (see Supplemental Material).

**Figure 5:**
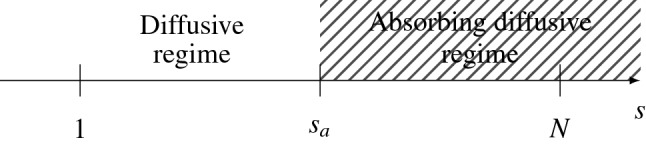
Schematic representation of the weakly and strongly absorbing diffusive regimes with length scales $s$ and $s_a$. $s= N$ denotes the usual transition to localization in a lossless medium.

The strength of absorption, whether strong or weak, will result in different transport regimes. In the very strong absorbing regime, the wave is rapidly damped before it experiences significant scattering. The conductance of a slab with length *L* is then $g = N \exp {(-L/\ell _a )}$, with $\ell _a \equiv 1/2k\gamma$ the ballistic absorption length. For weaker absorption such that $\ell _a \gg \ell$, multiple scattering will occur, allowing diffusive transport or transition to localization^30^. The impact on diffusion and localization of an absorbing medium has been extensively studied by Brouwer^31^, who generalized the Dorokhov–Mello–Pereyra–Kumar (DMPK) equation^21,32^ and derived the conductance

6 $$\begin{aligned} g_B (s,s_a ) = \dfrac{N}{s_a \sinh \left( \dfrac{s}{s_a }\right) +1}, \end{aligned}$$

where $s_a \equiv \xi _a/\ell$, with $\xi _a = \sqrt{\ell \ell _a /2}$ the diffusive absorption length. In the weakly absorbing diffusive regime ($s\ll s_a \ll N$, see Fig. 5), the conductance follows the Ohmic behavior predicted by the DMPK theory in conservative systems:

7 $$\begin{aligned} g_B (s,s_a ) \simeq g_D (s) = \dfrac{N}{s+1}. \end{aligned}$$

For stronger absorption, the absorbing diffusive regime ($s_a \ll s \ll N$) is characterized by an exponential decay of the conductance as

8 $$\begin{aligned} g_B (s,s_a ) \simeq \dfrac{2N}{s_a } e^{-s/s_a }. \end{aligned}$$

This absorption-induced exponential decrease makes it difficult to establish a clear threshold between diffusive and localized regimes. Other criteria based on the relative variations of the conductance are necessary^14^.

### A scaling model for the scattering with absorption

Now we consider the complete symmetric SBS. A scaling model is proposed in^12^ to compute the average conductance of the symmetric system in the lossless case. It is written as a series resistance model,

9 $$\begin{aligned} \frac{1}{\langle g(s,\tau )\rangle } = \frac{1}{g_D (s)} + \frac{1}{g_E (s,\tau )}, \end{aligned}$$

that involves the diffusive conductance of the slab, $g_D (s) = N/(s+1)$, and an “enhancement” conductance, $g_E (s,\tau )$, which encapsulates the combined effects of the barrier and symmetry. From the known limit cases, namely, $\langle g(s,1)\rangle = g_D (s)$ and $\langle g(0,\tau )\rangle = N \tau$, and a numerical analysis, we can write the latter as

10 $$\begin{aligned} g_E (s,\tau ) = \left( 1+\frac{\tau _c }{1-\tau _c }s\right) \frac{\tau }{1-\tau } N, \end{aligned}$$

where $\tau _c \simeq 0.4$ is a threshold value for the reduced transmittance of the barrier, above which no enhancement is observed. Eqs. (9, 10) accurately describe the symmetry-induced increase of the conductance, providing that the medium is lossless. This solution can be improved to take into account the absorption in the slabs.

**Figure 6:**
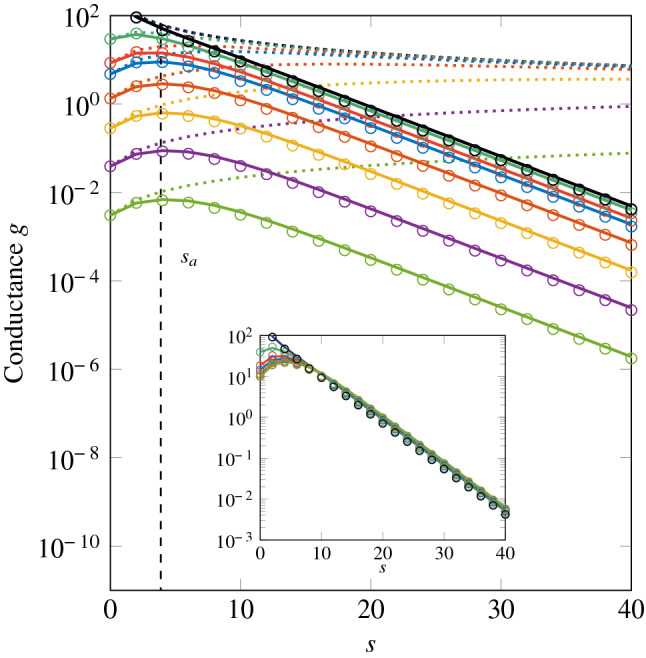
Averaged conductance of the absorbing symmetric SBS, as a function of the length of the system (in mean free path unit: $s\equiv L/\ell$), for various values of the barrier transmittance. Colored circles: RMT with $N = 300$ and $s_a = 3.87$, averaged over 100 iterations (See Supplemental Material). Solid colored lines: scaling model, Eq. (11). Dotted colored lines: Lossless conductance, as given by Eq. (9). Data in black (lines and circles) correspond to the configuration without barrier; in this case, the conductance is given by Eq. (6). Inset: product $\langle g(s,s_a ,\tau )\rangle \alpha (s_a ,\tau )$, showing that the shift between the data for large $s$ is given in the scaling model by the corrective factor $\alpha (s_a ,\tau )$. The barrier transmittances can be read at the y-intercepts of Fig. 6 as $\langle g(s=0,\tau )\rangle = N \tau$.

At first glance, it may seem sufficient to substitute $g_D$ for $g_B$ in Eq. (9). In fact it proves inaccurate. Indeed, such a simple resistance series model would imply that, at large $s$, $g_B ^{-1}$ dominates over $g_E ^{-1}$ and the overall conductance then tends towards a limit that is independent of the strength barrier $\tau$. This is what is observed in the lossless case (see Fig. 4 of Ref.^12^ and Fig. 6 below), but not when absorption is taken into account (Fig. 6). Thus we introduce an empirical corrective factor $\alpha$ and write the overall average conductance such that

11 $$\begin{aligned} \frac{1}{\langle g(s,s_a ,\tau )\rangle } = \frac{\alpha (s,s_a ,\tau )}{g_B (s,s_a )} + \frac{1}{g_E (s,\tau )}. \end{aligned}$$

Numerical computations show that this factor is independent of $s$ and may be simply written as

12 $$\begin{aligned} \alpha (s_a ,\tau ) = 1 + \frac{1}{2 s_a ^2 \tau }. \end{aligned}$$

This solution also ensures the consistency of Eq. (11) with Eq. (9) in the lossless limit $s_a \rightarrow \infty$.

Comparisons of this scaling model with RMT simulations, for various values of the diffusion length scale, $s_a$, and barrier strength, $\tau$, shows a very good agreement (see examples in Fig. 6 for a strong absorption, $s_a = 3.87$. The computation of absorbing transport with RMT and the determination of $s_a$ are explained in the Supplemental Material). As absorption occurs only in the slabs, the starting point of the curves at $s= 0$ is the transmittance $N \tau$ of the barrier, as in the lossless case (dotted lines). Then the curves deviate from this limit case to rapidly drop when $s> s_a$. The asymptotic behaviour at large $s$ is given by the Brouwer’s conductance Eq. (6) (black solid line) and the corrective factor $\alpha (s_a ,\tau )$ gives the shift between the asymptotics of each curve (see inset).

**Figure 7:**
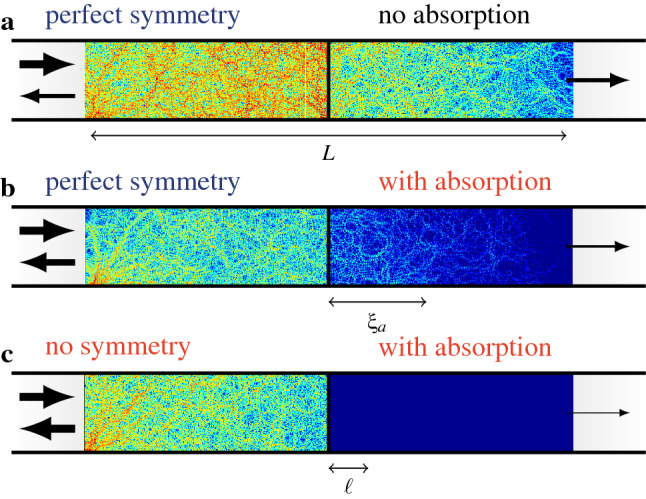
Persistence of the symmetry-induced conductance enhancement in an absorbing SBS. **(a)** Typical wavefield (full wave numerical computation) in a lossless symmetric SBS ($N = 50$, $\ell = 0.46$, $L = 6$). **(b)** Same as **(a)**, but with added background absorption in the diffusive slabs ($s_a = 2.2$). **(c)** Same as **(b)**, but without the left-right symmetry in the scatterers distribution.

Despite the very significant effect of losses, the symmetry-induced enhancement of the transmission is still observed (Fig. 7), and the evolution of conductance with $s$ shows that we can still expect an optimal length $s_opt$ of the system for which the ratio $\langle g\rangle /N\tau$ reaches a maximum value.

**Figure 8:**
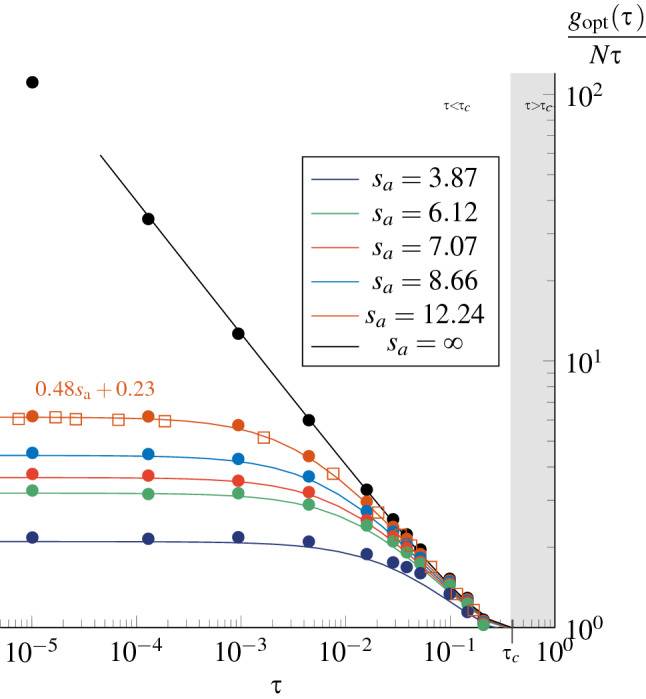
Variations of the maximum conductance enhancement as a function of the reduced barrier transmittance $\tau$, for various values of the diffusive absorption length $s_a$. Colored squares: full wave numerical simulations, averaged over 80 iterations of the disorder. Colored circles: RMT, averaged over 50 iterations. Solid lines: scaling model, Eq. (11). The saturation levels for $\tau \ll 1$ and finite $s_a$ are given by Eq. (14). A condition for achieving a transmission enhancement is that $\tau < \tau _c \simeq 0.4$^12^.

This can be simply found from Eq. (11), and the maximum enhancement thus obtained is in very good agreement with the numerical simulations, RMT and full wave (Fig. 8). Expectedly, the higher the absorption, the lower the maximum possible enhancement. But a remarkable result shown in this figure is the saturation of the conductance enhancement, due to absorption. While the increase of the enhancement in the lossless case in only limited by the transition to localization (see Supplemental Material), it is bounded, in the lossy case, by a maximum enhancement driven by the strength of absorption, that is, by $s_a$. Indeed, assuming $\tau \ll 1$, the derivation of the optimal length from Eq. (11) gives

13 $$\begin{aligned} s_opt \simeq 1.28 s_a - 1. \end{aligned}$$

We recall that Eq. (11), hence the result above, are valid under the assumption of diffusive transport ($1 \ll s, s_a \ll N$).

**Figure 9:**
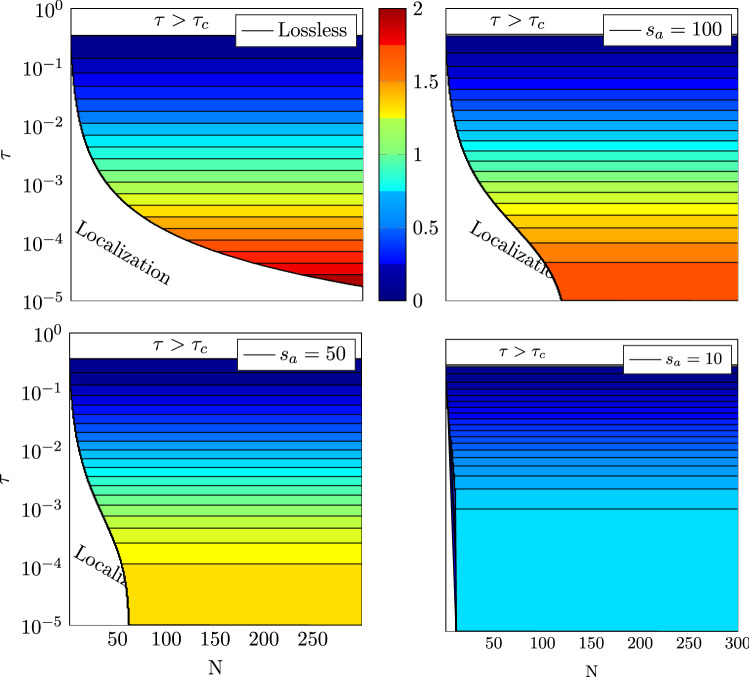
Maximum conductance enhancement $g_opt /N\tau$ that can be achieved for a given set of parameters $(N,\tau ,s_a )$, as deduced from the scaling model, Eq. (11).

Finally, the observations made on the effect of absorption on the transport through symmetric diffusive slabs can be summarized as done in Fig. 9, which shows how to practically get a particular enhancement. First, the range of possible values of the barrier transmittance $\tau$ and number of propagating modes *N* is limited by (i) the threshold value $\tau _c$, above which no enhancement is observed, and (ii) the transition to the localized regime (see Supplemental Material). Within these limits, regardless of the strength of absorption, the expected enhancement is independent of the number of propagating modes (note that it was already shown in^12^ for the lossless case, and $s_opt$ in Eq. (13) already appears as being independent of *N*). Finally, Fig. 9 shows the saturation of the conductance enhancement when decreasing $\tau$, and the maximum value is reached for $\tau$ values all the greater as the absorption is itself high. This maximum value, as well as the optimal length, depends only on the diffusive absorption length, as long as the barrier is sufficiently opaque:

14 $$\begin{aligned} \dfrac{g_opt }{N\tau } \simeq 0.48s_a + 0.23. \end{aligned}$$

## Conclusion

We have shown to what extent the significant enhancement of the transmission through lossless symmetric diffusive slabs is affected by defects of the symmetry in the scatterer distribution or by absorption. In the first case, rather than the amount of “defects” (the non-symmetrical pairs of scatterers), the distance from the barrier to the nearest defect appears as the limiting factor for the conductance enhancement. This could be particularly interesting for non-destructive testing applications, since the information on the position of a defect could therefore be available from a measurement of the conductance. When absorption occurs, the symmetry-induced enhancement, while weakened, is still observed, and this enhancement might even be a mean to experimentally quantify the absorption length, as its level is predominantly driven by the absorption.

## Supplementary information

Supplementary Information.
