# Supplementary material for: Sensitivity to losses and defects of the symmetry-induced transmission enhancement through diffusive slabs
Source: Sci Rep. 2020 Oct 6;10:16635. doi: 10.1038/s41598-020-73701-0 (PMC7538967; doi:10.1038/s41598-020-73701-0)
Supplement: Supplementary file 1 — Supplementary Information. [file 41598_2020_73701_MOESM1_ESM.pdf]

Supplementary Information to
Sensitivity to losses and defects of the symmetry-induced transmission enhancement through diffusive slabs

Élie Chéron, Simon Félix, Vincent Pagneux

June 18, 2020

1 RMT simulations with absorption

To solve the scattering problem, most of the numerical results shown are carried out using RMT simulations. Based on a method proposed by Verrier et al. [1], we consider a disorder slab, with length L, as being composed of $M \simeq L/\ell$ layers whose length is equal to the mean free path. The $2(N+N_{\mathrm{loss}})\times 2(N+N_{\mathrm{loss}})$ scattering matrices, modeled by elements of the Dyson's circular orthogonal ensemble (COE), fulfills the properties of reciprocity (symmetry) and energy conservation (unitarity). They are then reduced to $2N \times 2N$ matrices and the scattering matrix of the full slab is constructed iteratively from the M reduced matrices. The diffusive absorption parameter s_{a} is obtained by fitting the averaged conductance as a function of s to the conductance derived from the generalized Dorokhov-Mello-Pereyra-Kumar (DMPK) equation in the absorbing diffusive regime (Fig. 1). By making several fits, we come to the conclusion that the diffusive absorption length depends on N and N_{loss} such that

$$s_{\mathrm{a}} \simeq \sqrt{\frac{N}{2N_{\mathrm{loss}}}}. \tag{1}$$

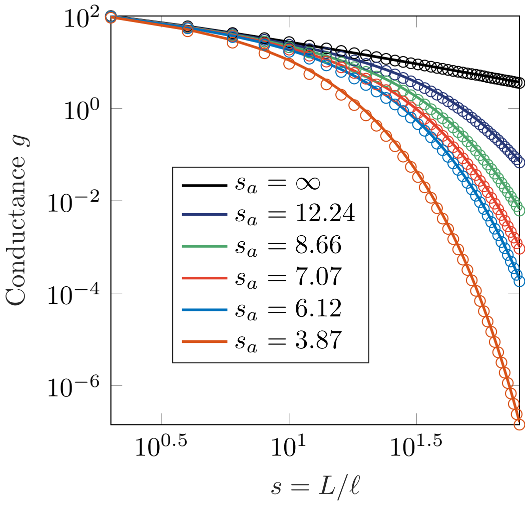

Figure 1: Colored circles: averaged conductance as a function of s obtained numerically using RMT simulations over 50 iterations with $N = 300$ and $N_{\mathrm{loss}} = 0, 1, 2, 2, 3, 4, 10$. Colored lines: fits from the conductance predicted by the Dorokhov-Mello-Pereyra-Kumar theory using s_{a} as the only fitting parameter.

2 Enhanced transmission with heterogeneous absorption

In the paper, for the sake of simplicity, we consider absorption as being uniform in the slab (background and scatterers). The purpose of this section is to extend the results to the case of heterogeneous absorption, namely absorbing scatterers in a non-absorbing background medium. This is accounted for in Eq. (5) of the paper by considering a non uniform function $\gamma(x, y)$ and using full wave numerics to compute the conductance.

Figure 2 shows, as does Fig. (6) of the paper for homogeneous absorption, the evolution of the averaged conductance with the length s of a symmetric "slab-barrier-slab" (SBS) system, for various values of the barrier transmittance. Here, absorption only occurs in the scatterers, not in the background medium, and the absorption length is $s_a = 8.38$. This value, as well as the mean free path ℓ, are obtained by fitting the numerical data for a slab without barrier with the "Brouwer" conductance, Eq. (6). The results shows that the scaling model, though built in the simple assumption of homogeneous absorption, still accurately recovers the exact full wave solution. A consequence is that, in that case too, the optimal length and conductance are mainly driven by the absorption length s_a, for sufficiently opaque barriers, as illustrated in Fig. 2 (inset).

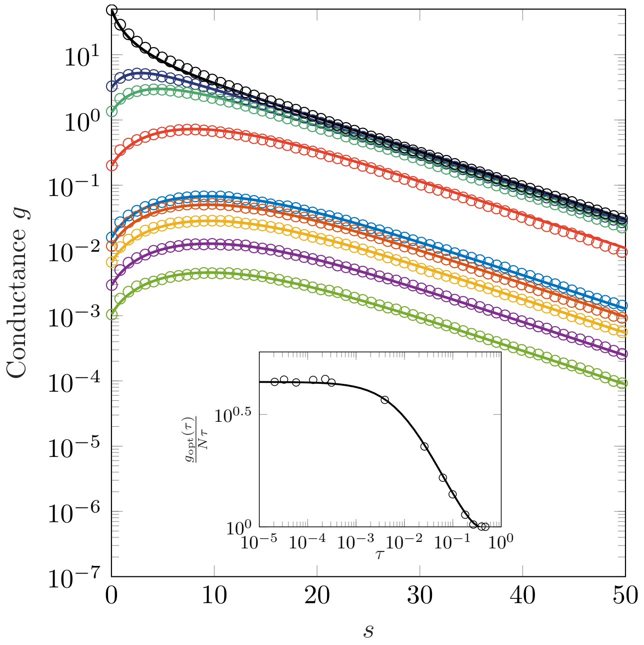

Figure 2: Averaged conductance of the absorbing symmetric SBS, as a function of the length of the system (in mean free path unit: $s \equiv L/\ell$), for various values of the barrier transmittance, τ. The barrier transmittances can be read at the y-intercepts, as $\langle g(s = 0, \tau)\rangle = N\tau$. Colored circles: full wave numerics with $N = 50$ and $s_a = 8.38$, averaged over 80 iterations. Solid colored lines: scaling model. Inset: conductance enhancement $g_{opt}/N\tau$ as function of the barrier transmittance τ, to be compared with Fig. 8 of the paper.

3 Enhanced transmission induced by symmetric localized slabs

Localization is a phenomenon purely related to multiple wave interferences that occurs in situations where the length of the system, L, is longer than the localization length $\xi = N\ell$. Solving the DMPK equation in the limit $s \gg N$ shows that the conductance fluctuations become large and the transmission drops exponentially with the length of the medium: $g_{loc} = \exp(-L/\xi)$ [2, 3]. We consider in this section the fate of the symmetry-induced conductance enhancement, originally shown in the diffusive regime [4], when reaching the localized regime.

In the diffusive regime, the maximum conductance enhancement $g_{\rm opt}/N\tau$, with $g_{\rm opt} = g(s_{\rm opt}, \tau)$, was shown to scale as the inverse square root of the barrier transmittance (Fig. 3). Also, the optimal length $s_{\rm opt}$ increases with the strength of the barrier, that is, when decreasing its transmittance τ. Therefore, below a certain value of the transmittance, the assumption of diffusive transport, $s \ll N$, might be no longer valid, hence the $\sqrt{1/\tau}$ scaling for the conductance enhancement. This is indeed what is observed (Fig. 3): the increase of $g_{\rm opt}$ with decreasing τ reaches a limit when approaching the localization regime. This limit value, which gives the highest possible enhancement, increases with N, that is, with the frequency. In conclusion, in the localized regime, the transport in a quasi-1D waveguide still experiences a symmetry-induced conductance enhancement. This enhancement, rather than being driven by τ as in the diffusive regime, is predominantly driven by N.

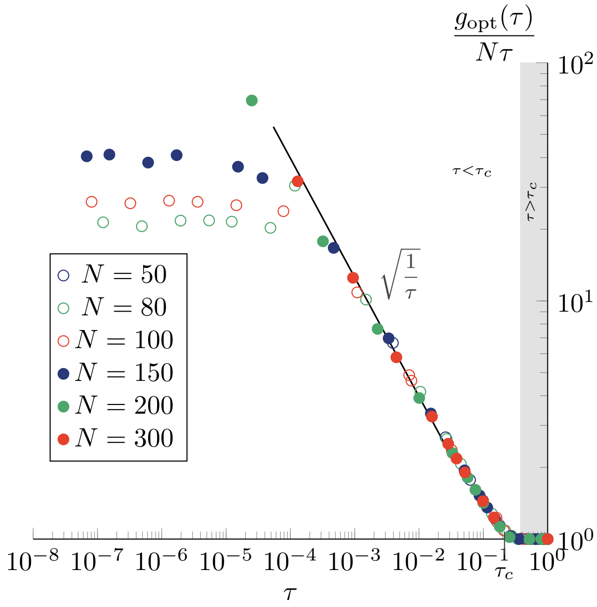

Figure 3: Variations of the maximum conductance enhancement as a function of the reduced barrier transmittance τ, for various values of N. Colored plain and open circles: RMT, averaged over 50 iterations. Note that, because of the large fluctuations of the conductance in the localized regime, averaging the conductance itself is no longer relevant. Therefore, the average value of $\log(g)$ is used instead to determine $g_{\rm opt}$ from the numerical data. Solid lines: scaling model given in [4]. The grey zone shows the range $\tau_c < \tau < 1$ where no conductance enhancement occurs.

Let us now have a closer look at the particular case of a purely 1D transport, $N = 1$. Then a direct transition occurs from the ballistic to the localized regime, as soon as s is larger than 1. The conductance of an ordinary SBS system (without symmetry) reads as the product of the localized conductance $g_{\rm loc}$ of the L-length slab and the transmittance of the barrier [2]:

$$\langle \log g(s) \rangle = \log g_{\rm loc}(s) + \log \tau. \tag{2}$$

In Fig. 4, we compare the cases of ordinary and symmetric disorder, with and without barrier: all follow the equation above. This means that symmetry has no significant effect on transmission when only one mode is propagating. The conductance enhancement can only be achieved with multimodal propagation.

References

[1] N. Verrier, L. Depraeter, D. Felbacq, and M. Gross. Measuring enhanced optical correlations induced by transmission open channels in a slab geometry. *Phys. Rev. B*, 93:161114, 2016.

[2] C. W. J. Beenakker. Random-matrix theory of quantum transport. *Rev. Mod. Phys.*, 69:731, 1997.

[3] S. Rotter and S. Gigan. Light fields in complex media: Mesoscopic scattering meets wave control. *Rev. Mod. Phys.*, 80:015005, 2017.

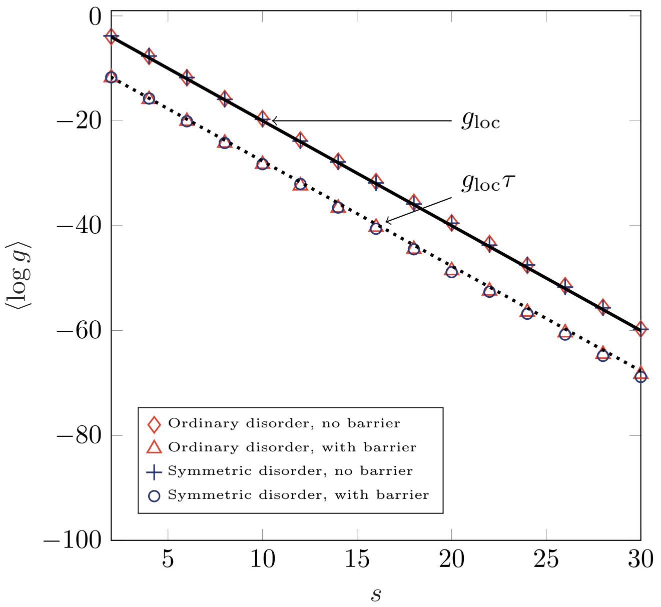

Figure 4: Averaged conductance as a function of s using RMT simulations over 50 iterations with $N = 1$. Red symbols : Ordinary disorder without (diamonds) and with (triangles) an opaque barrier of transmittance $\log(\tau) = -7.7$. Blue symbols : Symmetric disorder without (crosses) and with (circles) an opaque barrier of transmittance $\log(\tau) = -7.7$. All configurations follow the typical averaged conductance given by Eq. (2) (plain and dotted lines). No enhancement due to spatial symmetry is observed.

[4] E. Chéron, S. Félix, and V. Pagneux. Broadband-enhanced transmission through symmetric diffusive slabs. *Phys. Rev. Lett.*, 122:125501, 2019.
